# Supplementary material for: Voltage‐dependent activation of Rac1 by Nav1.5 channels promotes cell migration
Source: J Cell Physiol. 2019 Oct 15;235(4):3950–72. doi: 10.1002/jcp.29290 (PMC6973152; doi:10.1002/jcp.29290)

**Supplementary Figure Legends**

**Figure S1.** Effects of sodium citrate and phenytoin on the membrane potential. (A) V_m_ in control physiological saline solution, after perfusion with the vehicle for TTX (148 µM sodium citrate, pH = 4.8) and following washout. Solid line, mean; gray shading, SEM (n = 13). (B) Quantification of V_m_ over the last 5 s in control, sodium citrate, and washout (n = 13). (C) Representative trace showing the inhibitory effect of phenytoin (100 µM) on Na^+^ current, and recovery after washout. The cell was held at -120 mV for 250 ms before depolarizing to -10 mV for 50 ms. (D) Expanded view of persistent Na^+^ current 40-45 ms following onset of depolarization. (E) Quantification of the normalized transient Na^+^ current elicited by depolarizing to -10 mV from a holding potential of -120 mV (n = 9). (F) Quantification of the normalized persistent Na^+^ current 40-45 ms after depolarizing to -10 mV from a holding potential of -120 mV (n = 4). (G) Quantification of the normalized transient Na^+^ current elicited by depolarizing to -10 mV from a holding potential of -80 mV (n = 4). (H) Quantification of the normalized persistent Na^+^ current 40-45 ms after depolarizing to -10 mV from a holding potential of -80 mV (n = 3). (I) V_m_ in control physiological saline solution, after phenytoin (100 µM) treatment and following washout. Solid line, mean; gray shading, SEM (n = 12). (J) Quantification of V_m_ over the last 5 s in control, phenytoin, and washout (n = 12). (K) Quantification of V_m_ over the last 5 s in control, NaOH (75 µM; vehicle for phenytoin) and washout (n = 9). Data are mean and SEM. *P < 0.05; **P < 0.01; ***P < 0.001; repeated measures ANOVA with Tukey test.

**Figure S2.** Effects of veratridine on Na^+^ current and NMDG on membrane potential. (A) I-V relationship for transient Na^+^ current in control physiological saline solution and following perfusion of veratridine (100 µM) (n = 6). (B) I-V relationship for persistent Na^+^ current (defined as mean current density 45-50 ms following onset of depolarization; n = 6). Data are mean and SEM. Consistent with previous reports (Ulbricht, 1969, 1998), veratridine caused a small reduction in the transient peak Na^+^ current density but increased the peak persistent Na^+^ current density. (C) V_m_ in control physiological saline solution, after extracellular Na^+^ replacement with N-methyl-D-glucamine (NMDG) and following washout. Solid line, mean; gray shading, SEM (n = 7). (D) Quantification of V_m_ over the last 5 s in control, NMDG, and washout (n = 7).

**Figure S3.** Tetrodotoxin and NS-1619 do not affect the intracellular Ca^2+^ level. (A) Intracellular Ca^2+^ level (340/380 ratio) following perfusion with TTX (30 µM). Solid line, mean; gray shading, SEM (n = 55). (B) 340/380 ratio over the last 30 s in control, TTX and washout (n = 3). (C) Intracellular Ca^2+^ level (340/380 ratio) following pre-treatment with TTX (30 µM) for 48 h (n = 55). (D) 340/380 ratio over the last 30 s of TTX and washout (n = 3). (E) Intracellular Ca^2+^ level (340/380 ratio) following perfusion with NS-1619 (1 µM). Solid line, mean; gray shading, SEM (n = 40). (F) 340/380 ratio over the last 30 s in control, NS-1619 and washout (n = 3). Ionomycin was used at the end of all experiments as a positive control confirming sensitivity of the Ca^2+^ indicator. Data are mean and SEM.

**Figure S4.** Effect of tetrodotoxin and NS-1619 on proliferation and invasion. (A) Proliferation (quantified as number of cells using the MTT assay) following treatment for 24 h with NS-1619 (1 µM, 40 µM) or vehicle (n = 3). (B) Matrigel invasion ± NS-1619 (1 µM) or TTX (30 µM), normalized to control (n = 4). (C) Matrigel invasion ± NS-1619 (40 µM), normalized to control (n = 3). Data are mean and SEM. *P < 0.05; **P < 0.01; repeated measures ANOVA with Tukey test.

**Figure S5.** Dose-dependent effect of EHT1864 on cell morphology. (A) Circularity of cells after treatment with EHT1864 (0.5-10 µM) or vehicle for 3 h (n ≥ 277). (C) Feret’s diameter (µm) of cells after treatment with EHT1864 (0.5-10 µM) or vehicle for 3 h (n ≥ 277). Data are mean and SEM.

**References**

Ulbricht, W. (1969). The effect of veratridine on excitable membranes of nerve and muscle. *Ergebnisse der Physiologie, Biologischen Chemie und Experimentellen Pharmakologie, 61*, 18-71. Retrieved from <http://www.ncbi.nlm.nih.gov/pubmed/4903416>.

Ulbricht, W. (1998). Effects of veratridine on sodium currents and fluxes. *Reviews of Physiology Biochemistry and Pharmacology, 133*, 1-54. Retrieved from <http://www.ncbi.nlm.nih.gov/pubmed/9600010>.

Figure S1
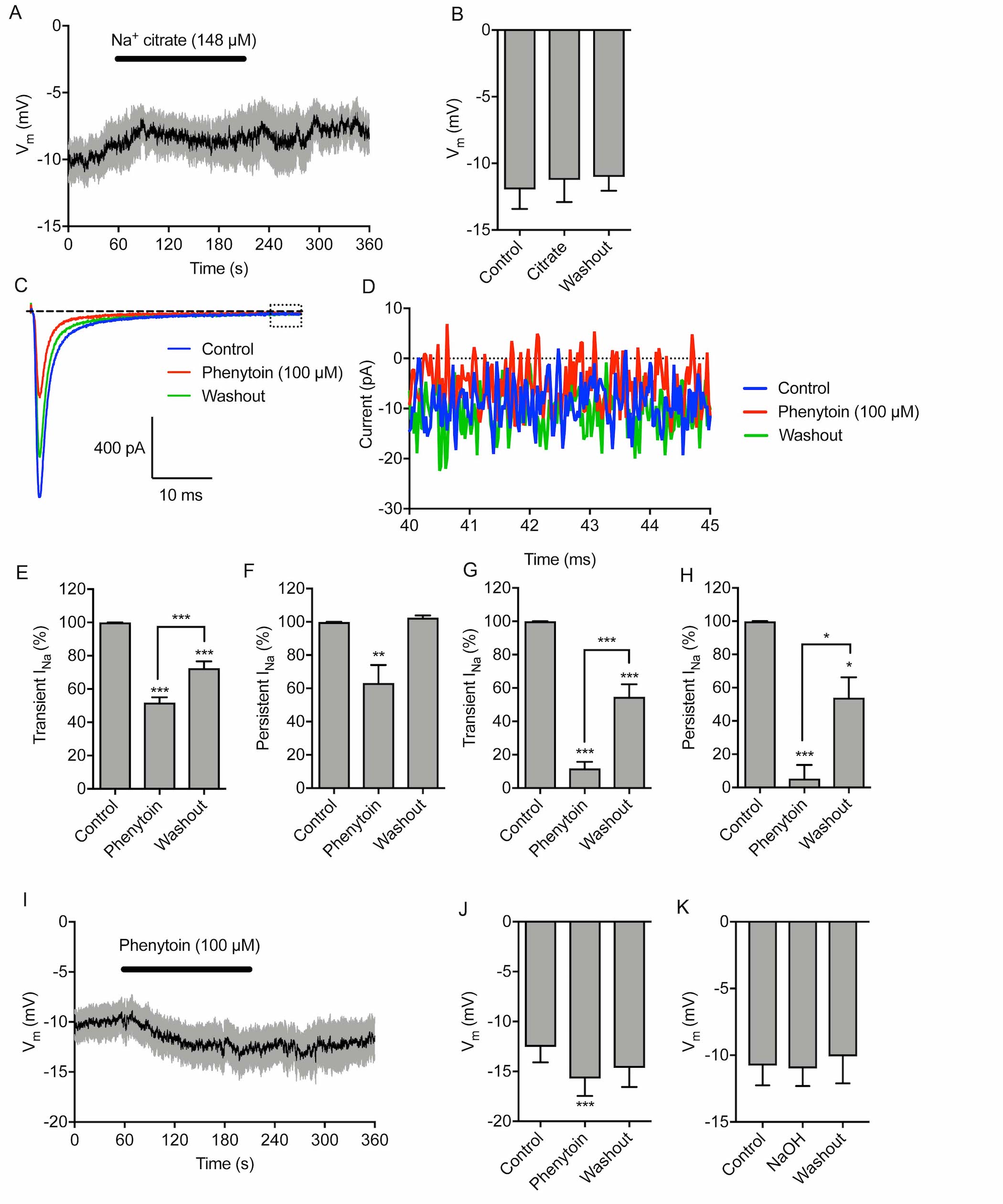


Figure S2
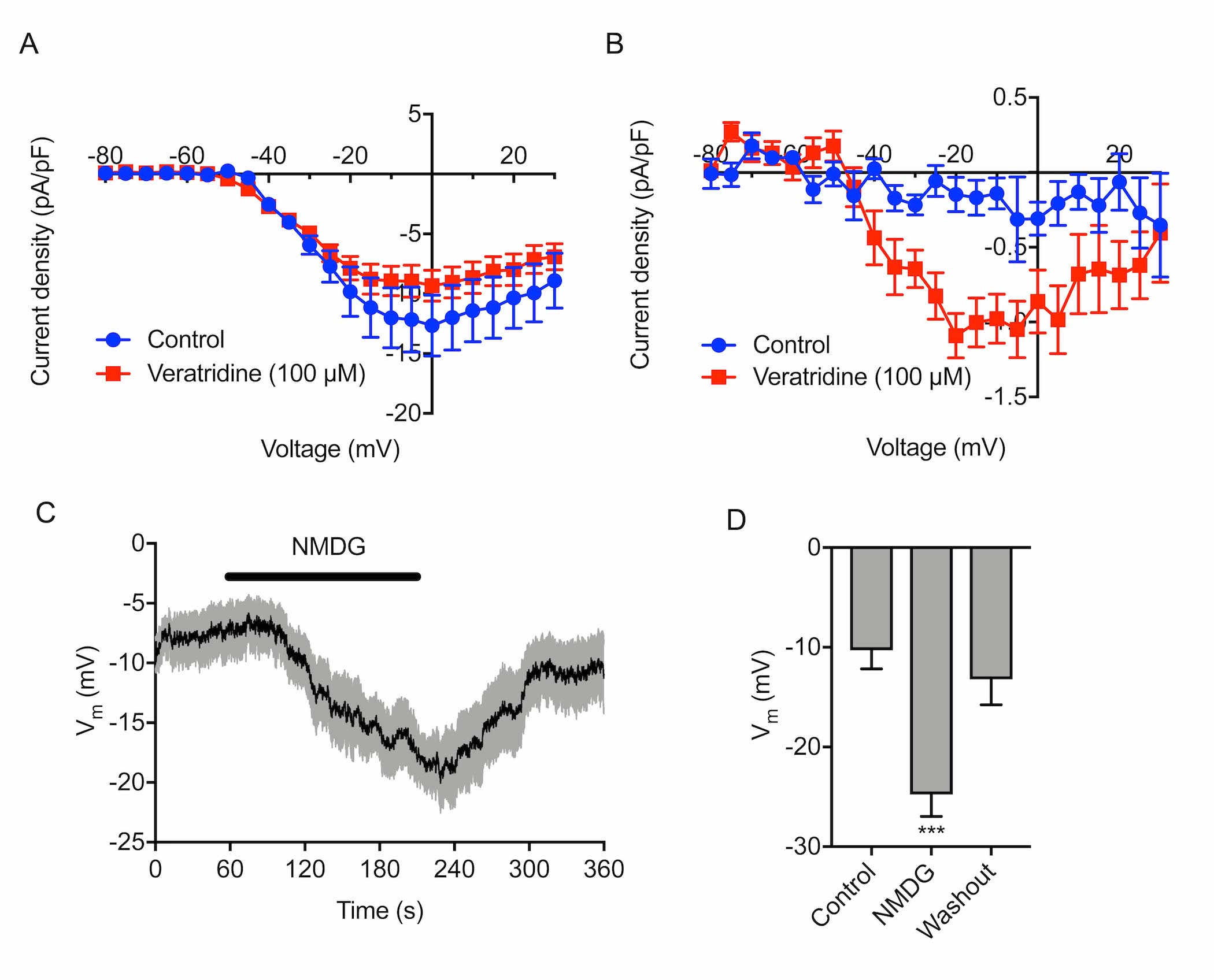


Figure S3


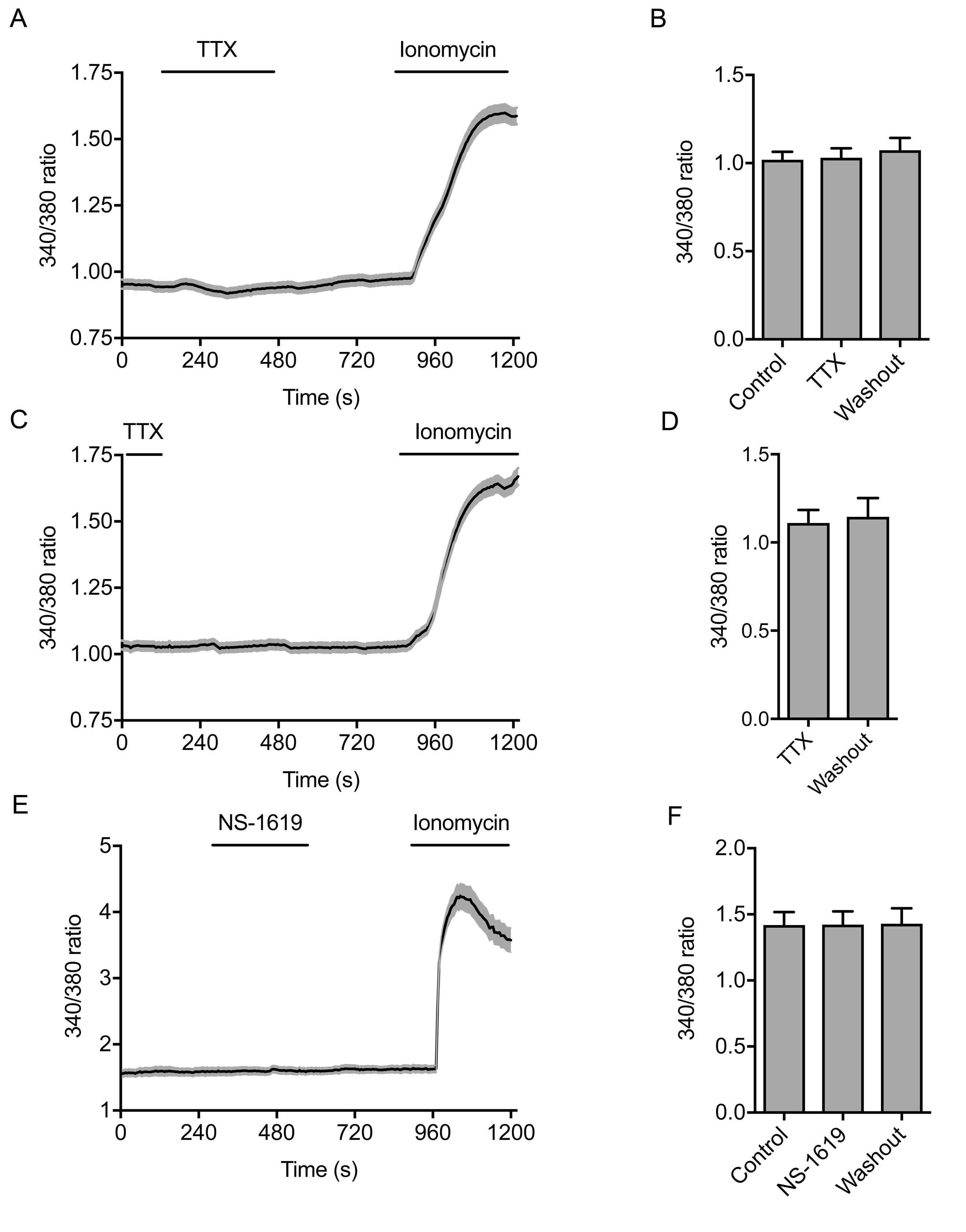


Figure S4
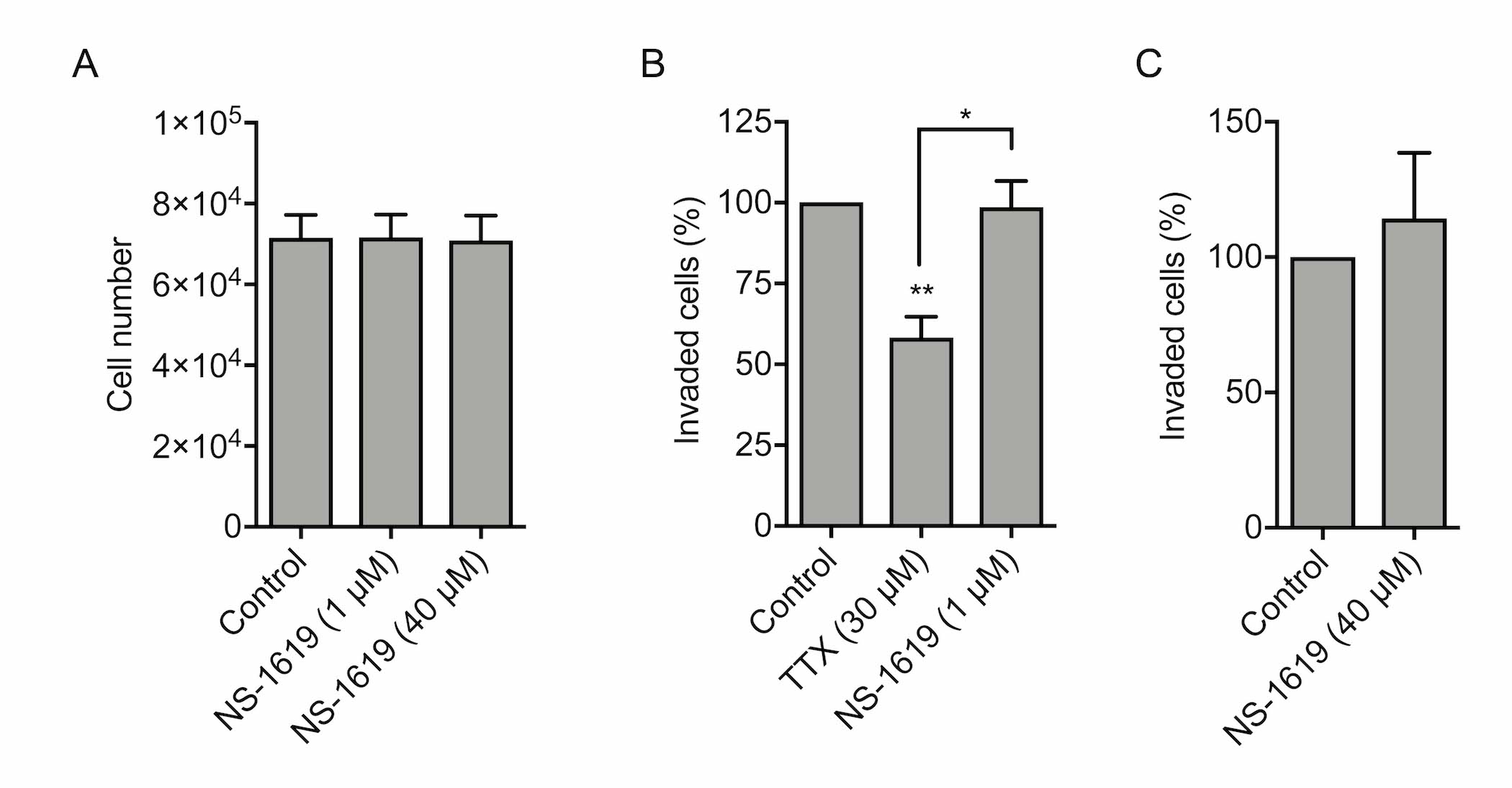


Figure S5
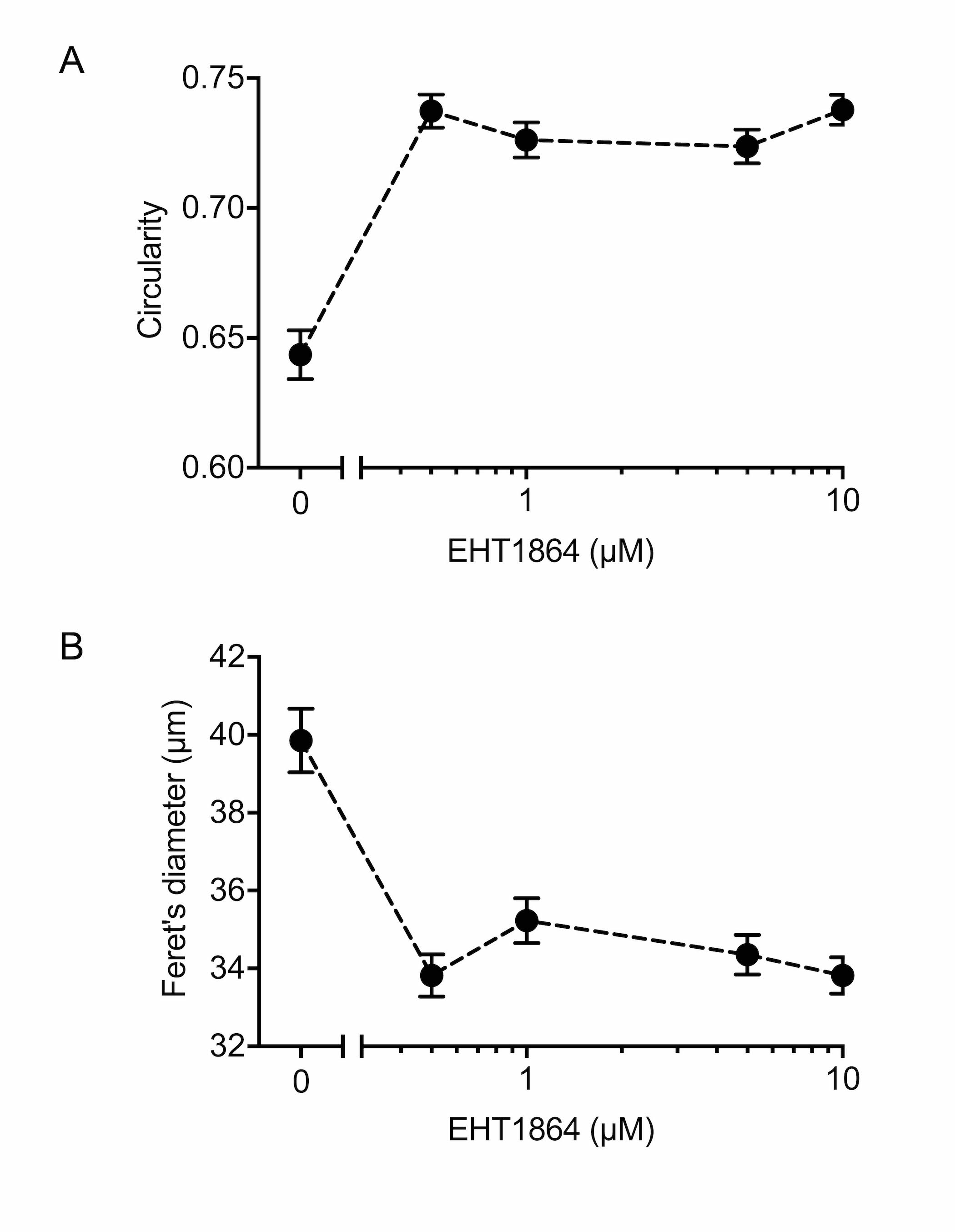

Supplement: Supplementary file 1 — Supplementary information [file JCP-235-3950-s001.docx]
